# Inflammatory and Nutritional Indices as Prognostic Markers in Locally Advanced Gastric Cancer Treated with Neoadjuvant FLOT: A Retrospective Multicenter Study

**DOI:** 10.3390/jcm15072574

**Published:** 2026-03-27

**Authors:** Süleyman Tuna Yolcu, Murat Günaltılı, Emine Ayaz Yolcu, Süleyman Sami Güzel, Nilay Şengül, Muhammed Mustafa Atcı, Kubilay Karaboyun, Gökmen Umut Erdem, Özkan Alan, Nebi Serkan Demirci

**Affiliations:** 1 Department of Internal Medicine, Cerrahpaşa Faculty of Medicine, Istanbul University-Cerrahpaşa, Istanbul 34098, Turkey; 2 Division of Medical Oncology, Department of Internal Medicine, Cerrahpaşa Faculty of Medicine, Istanbul University-Cerrahpaşa, Istanbul 34098, Turkeysuleymanguzel34@hotmail.com (S.S.G.); 3 Department of Medical Oncology, University of Health Science, Istanbul Training and Research Hospital, Istanbul 34098, Turkey; 4 Department of Medical Oncology, University of Health Sciences, Professor Doctor Cemil Tascioglu City Hospital, Istanbul 34384, Turkey; 5 Division of Medical Oncology, Department of Internal Medicine, Tekirdağ Namık Kemal University, Tekirdağ 59030, Turkey; 6 Department of Medical Oncology, University of Health Sciences, Basaksehir Cam and Sakura City Hospital, Istanbul 34480, Turkey

**Keywords:** gastric cancer, pathological response, FLOT regimen, inflammatory and nutritional indices, CEA/albumin ratio

## Abstract

**Background/Objectives**: Neoadjuvant chemotherapy is the standard of care for patients with locally advanced gastric cancer. Inflammatory and nutritional indices have been proposed as potential prognostic biomarkers in this setting. This study aimed to evaluate their association with pathological response and relapse-free survival (RFS) in patients with locally advanced gastric cancer treated with the perioperative FLOT regimen. **Methods**: This multicenter retrospective study included 120 patients treated with perioperative FLOT between 2018 and 2022. Pathological response was assessed using the Becker regression grading system. Pretreatment inflammatory and nutritional biomarkers were calculated from baseline data. Association with pathological responses and RFS were analyzed using logistic regression, Kaplan–Meier estimates, and Cox models. **Results**: Pathological response was strongly associated with higher radiologic and R0 resection rates and lower recurrence (all *p* < 0.001). None of the biomarkers correlated significantly with pathological response. Pathological response was the strongest prognostic factor for RFS (*p* < 0.001), while age (*p* = 0.011) and histologic subtype (*p* = 0.004) were also independent predictors. The CEA/albumin ratio showed a trend toward significance (HR 1.805; 95% CI 0.918–3.551; *p* = 0.087), and patients with lower ratios had longer RFS (median not reached vs. 15.8 months, *p* = 0.014). **Conclusions**: Pathological response remains the most powerful prognostic factor in locally advanced gastric cancer treated with perioperative FLOT. Although inflammatory and nutritional indices alone may not predict treatment response, the CEA/albumin ratio demonstrates potential prognostic value for RFS. Larger prospective studies with standardized cut-off values are warranted to validate these findings and to further explore the dynamic prognostic role of immunonutritional markers.

## 1. Introduction

Gastric cancer ranks fifth among the most common malignancies worldwide and is the fourth leading cause of cancer-related mortality [1]. Because it usually remains asymptomatic in the early stages, a substantial proportion of patients are diagnosed at a locally advanced stage, where surgery alone is insufficient to achieve a cure. Even after achieving R0 resection, recurrence rates can reach up to 60%, underscoring the need for multimodal treatment approaches [2]. In operable gastric cancer, perioperative chemotherapy has demonstrated a survival advantage over surgery alone. For many years, the perioperative epirubicin, cisplatin, and fluorouracil (ECF) regimen was widely used for this purpose [3]. The FLOT4 trial subsequently established the superiority of the perioperative FLOT regimen (5-fluorouracil, leucovorin, oxaliplatin, docetaxel) over ECF, making FLOT the current standard of care [4]. Furthermore, the MATTERHORN trial, which investigated the addition of durvalumab to perioperative FLOT, reported a significant improvement in the pathological complete response rates with an acceptable safety profile [5].

Despite comparable disease stages and treatment regimens, patients demonstrate substantial variability in pathological response and survival following FLOT therapy. This heterogeneity highlights the need for reliable biomarkers to predict treatment response. Although clinicopathological factors (e.g., histological type and stage) provide partial guidance, the predictive and prognostic values of biological markers have been increasingly investigated. Chronic inflammation, nutritional impairment, and immune system alterations play pivotal roles in the development and progression of gastric cancer. Inflammatory processes promote tumor progression and metastasis within the tumor microenvironment through the action of cytokines, chemokines, and immune cell infiltration [6,7]. Malnutrition compromises immune competence, reduces treatment tolerance, and adversely affects survival [8]. Although both innate and adaptive components of the immune system are crucial for recognizing and eliminating tumor cells, tumor-associated chronic inflammation may foster immunosuppression and immune evasion. Thus, a combined assessment of inflammatory and nutritional parameters may be critical for understanding gastric cancer biology and predicting prognosis [9,10].

Several inflammation- and nutrition-based indices derived from readily available laboratory parameters have been proposed to reflect systemic inflammation and nutritional status of patients. These include the neutrophil-to-lymphocyte ratio (NLR), platelet-to-lymphocyte ratio (PLR), prognostic nutritional index (PNI), systemic immune-inflammation index (SII), hemoglobin-albumin-lymphocyte-platelet score (HALP), neutrophil-to-albumin ratio (NAR), hemoglobin-to-red cell distribution width (RDW) ratio (HRR), and RDW-to-albumin ratio (RAR). These markers have been reported to be associated with prognosis and treatment response, particularly in locally advanced or metastatic gastric cancer [11,12,13,14,15,16]. However, some studies have failed to demonstrate a significant correlation between these indices and treatment outcomes [17,18,19,20]. In addition, ratio-based indices, such as the carcinoembryonic antigen-to-albumin ratio (CEA/albumin), have been suggested to be associated with pathological response [21,22].

In the current literature, only a limited number of studies have investigated inflammatory and nutritional indices in relation to both pathological response and RFS in patients treated with perioperative FLOT [23,24]. Therefore, this study aimed to evaluate the association between pathological response and RFS and to explore the prognostic and predictive significance of inflammatory and nutritional scores (NLR, PLR, PNI, SII, HALP, NAR, HRR, and RAR) as well as ratio-based composite index (CEA/albumin) in patients with locally advanced gastric cancer receiving perioperative FLOT therapy.

## 2. Materials and Methods

### 2.1. Study Design and Patient Population

This retrospective, multicenter study included 120 patients diagnosed with localized and locally advanced gastric cancer who received perioperative FLOT chemotherapy between January 2018 and December 2022 at four tertiary referral centers in Turkey. Eligible patients had histologically confirmed gastric adenocarcinoma staged according to the American Joint Committee on Cancer (AJCC) Staging Manual, 8th edition, with inclusion criteria requiring cT2 or node-positive disease [25]. The perioperative FLOT regimen consisted of four cycles before surgery and four cycles after surgery, comprising docetaxel (50 mg/m^2^, intravenously), oxaliplatin (80 mg/m^2^, intravenously), leucovorin (200 mg/m^2^, intravenously), and fluorouracil (2600 mg/m^2^ as a 24-h continuous intravenous infusion). Patients younger than 18 years of age, those with metastatic disease at diagnosis, incomplete clinical data, inability to complete all four cycles of neoadjuvant FLOT, failure to undergo surgical resection after neoadjuvant treatment, and patients with a performance status greater than 2 according to the Eastern Cooperative Oncology Group (ECOG) were excluded.

### 2.2. Data Collection and Variables

Clinicopathological and laboratory data were retrospectively retrieved from the electronic medical records and institutional archives. Baseline variables included demographic characteristics (age and sex), ECOG performance status, tumor localization, histological subtype, differentiation grade, Lauren classification, and clinical TNM stage according to the American Joint Committee on Cancer (AJCC), 8th edition. Additional variables included radiological response, surgical margin status (R0/R1), and disease relapse.

Inflammatory and nutritional indices were calculated using pretreatment peripheral blood counts and serum biochemistry values obtained before the administration of the first cycle of neoadjuvant FLOT chemotherapy.

The following indices were derived using previously validated formulas:

- Neutrophil-to-lymphocyte ratio (NLR): neutrophil count/lymphocyte count
- Platelet-to-lymphocyte ratio (PLR): platelet count/lymphocyte count
- Prognostic Nutritional Index (PNI): albumin (g/L) + 5 × lymphocyte count (×10^9^/L)
- Systemic Immune-inflammation Index (SII): (platelet × neutrophil)/lymphocyte
- Hemoglobin, Albumin, Lymphocyte, and Platelet score (HALP): hemoglobin × albumin × lymphocyte/platelet
- Neutrophil-to-Albumin Ratio (NAR): neutrophil count/albumin
- Hemoglobin-to-RDW Ratio (HRR): hemoglobin/red cell distribution width
- RDW-to-Albumin Ratio (RAR): red cell distribution width/albumin
- CEA-to-Albumin Ratio: carcinoembryonic antigen/albumin
- CA19-9-to-Albumin Ratio: carbohydrate antigen 19-9/albumin

### 2.3. Pathological Response Assessment

Pathological tumor regression was evaluated according to the Becker regression grading system [26], which categorizes tumor regression based on the proportion of residual viable tumor cells relative to the total tumor bed. The grading system is defined as follows: TRG 1a, complete regression with no residual tumor cells; TRG 1b, subtotal regression with <10% residual tumor cells; TRG 2, partial regression with 10–50% residual tumor cells; and TRG 3, minimal regression with >50% residual tumor cells. For this study, patients with TRG 1a, 1b, or 2 were classified as non-TRG 3 (responders), whereas those with TRG 3 were classified as TRG 3 (non-responders). All histopathological evaluations were performed by experienced gastrointestinal pathologists at participating centers.

### 2.4. Follow-Up and Survival Analysis

Follow-up was calculated from the date of diagnosis to the date of the last follow-up or death. The primary endpoint was relapse-free survival (RFS), defined as the interval from the date of surgery to the first documented recurrence of the disease. Patients without recurrence were censored at the date of last follow-up, whereas those who died without recurrence were censored at the date of death. Overall survival (OS) was defined as the time from the initiation of the first cycle of neoadjuvant FLOT chemotherapy to death from any cause or last follow-up.

### 2.5. Ethical Considerations

This study was conducted in accordance with the principles of the Declaration of Helsinki. Ethical approval was obtained from the Ethics Committee of Istanbul University-Cerrahpaşa, Cerrahpaşa Medical Faculty, Turkey (approval number: E-83045809-604.01.01-590310; approval date: 10 January 2023). Informed consent for participation is not required as per local legislation, as confirmed by the Ethics Committee of Istanbul University-Cerrahpaşa, Cerrahpaşa Medical Faculty, due to the retrospective design of the study.

### 2.6. Statistical Analysis

All statistical analyses were performed using the SPSS software (version 27.0; IBM Corp., Armonk, NY, USA). The normality of the distribution of continuous variables was assessed using the Shapiro–Wilk test. Continuous variables are expressed as mean ± standard deviation when normally distributed or as median and range when not normally distributed. Categorical variables were summarized using counts and percentages. Between-group comparisons were performed using the independent samples *t*-test for normally distributed continuous variables and the Mann–Whitney U test for non-normally distributed variables. Categorical variables were compared using the chi-square test or Fisher’s exact test, as appropriate.

Receiver operating characteristic (ROC) curve analyses were initially conducted to identify the optimal cut-off values of inflammatory and nutritional indices for predicting pathological response. However, none of the markers demonstrated statistically significant discriminatory performance based on ROC analysis. Therefore, to avoid arbitrary threshold selection and to maintain statistical robustness, the variables were dichotomized according to the median values of the study cohort. The manuscript has been revised accordingly.

Univariate and multivariate logistic regression analyses were conducted to evaluate the factors associated with pathological response. Variables that were significant in the univariate analysis or deemed clinically relevant based on prior evidence were included in the multivariate model.

Relapse-free survival was estimated using the Kaplan–Meier method, and differences between groups were assessed using the log-rank test. Cox proportional hazards regression models were used to identify prognostic factors for RFS. Variables that were significant in the univariate analysis or considered clinically relevant were included in the multivariate model.

All statistical tests were two-sided, and *p*-values < 0.05 were considered statistically significant.

## 3. Results

### 3.1. Patient Characteristics

A total of 120 patients with gastric cancer who received perioperative FLOT chemotherapy were included in this multicenter study, comprising 10 patients from Cerrahpaşa Medical Faculty, 31 patients from Istanbul Training and Research Hospital, 9 patients from Tekirdağ Namık Kemal University, and 70 patients from Haseki Training and Research Hospital. The median age at diagnosis was 59 years (range, 26–86 years), and 71% of the cohort were male. The performance status was generally preserved, with 85% of patients having an ECOG score of 0 and 14% having a score of 1.

Tumor localization was most frequent at the cardia (35%) and antrum (31%), followed by the corpus (23%) and gastroesophageal junction (12%). Adenocarcinoma represented the majority of histological subtypes (75%), whereas 25% were signet-ring cell carcinomas. According to the Lauren classification, the intestinal type was the most common (64%), followed by diffuse/mixed (20%) and unknown (16%) tumors. At diagnosis, 91% of the patients had cT3–4 disease, and 87% were node-positive. Clinically, 84% of the patients presented with stage III disease, while 16% had stage II disease.

All patients received neoadjuvant FLOT chemotherapy and subsequently underwent surgery. However, 23 patients received more than four cycles of neoadjuvant FLOT chemotherapy because their operations were postponed due to the COVID-19 pandemic. A radiological response was observed in 72% of the overall cohort, and curative (R0) resection was achieved in 96% of patients. Complete radiological response was identified in 11 patients (%9.2). In addition, 31 patients (%25.8) had stable disease, while disease progression was observed in 3 patients (%2.5).

Pathological response assessment according to Becker’s classification divided the cohort into two groups: patients with a pathological response (non-TRG 3, n = 87) and those without (TRG 3, n = 33). The non-TRG 3 group exhibited significantly higher rates of radiological response (*p* < 0.001) and R0 resection (*p* = 0.001), along with a significantly lower relapse rate (*p* < 0.001). However, no statistically significant differences were observed between the two groups with respect to age, sex, clinical stage, histological subtype, or Lauren classification (all *p* > 0.05). The detailed distribution of clinicopathological characteristics for the entire cohort and according to the pathological response is presented in Table 1.

### 3.2. Inflammatory and Nutritional Markers in Relation to Pathological Response

ROC curve analysis was initially performed to determine the optimal cut-off values for inflammatory and nutritional indices in predicting pathological responses. However, none of the markers achieved a statistically significant area under the curve (AUC) to define clinically meaningful thresholds. Therefore, each marker was dichotomized based on the median value within the cohort for subsequent categorical analyses.

When comparing responders (non-TRG 3) and non-responders (TRG 3), no statistically significant differences were observed in the median values of NLR, PLR, PNI, SII, HALP, NAR, HRR, RAR, CEA/albumin ratio, or CA19-9/Albumin ratio (all *p* > 0.05). The detailed results are summarized in Table 2.

### 3.3. Factors Associated with Pathological Response

Logistic regression analyses were conducted to evaluate the predictors of pathological response. In univariate analyses, histologic subtype (*p* = 0.080), Lauren classification (overall *p* = 0.089), clinical T stage (*p* = 0.183), primary G-CSF prophylaxis (*p* = 0.084), RAR (*p* = 0.077), and CA19-9/Albumin ratio (*p* = 0.103) showed statistical or borderline associations.

Based on these findings, variables with univariate *p*-values < 0.10, along with clinically relevant factors, were included in the multivariate analysis. In this analysis, none of the variables were statistically significant. Histologic subtype demonstrated a trend toward association (OR: 0.383, 95% CI: 0.131–1.124, *p* = 0.081), while Lauren classification (overall *p* = 0.129), cT stage (*p* = 0.729), G-CSF prophylaxis (*p* = 0.073), RAR (*p* = 0.362), and CA19-9/Albumin ratio (*p* = 0.173) were not significant. The results of the univariate and multivariate logistic regression analyses are summarized in Table 3.

### 3.4. Factors Associated with Relapse-Free Survival

The median follow-up duration was 26.1 months (range, 4.3–55.9 months). During follow-up, disease relapse occurred in 49 patients (40.8%), of whom 4 were alive at the time of analysis. Additionally, 4 patients died without documented relapse. The median relapse-free survival (RFS) was not reached during the study period. A total of 49 death events were observed. The median overall survival was 49.0 months (95% CI: 40.15–57.85), as shown in Figure S1. The results of the univariate and multivariate Cox regression analyses are also presented in Table S1.

Cox regression analysis was performed to evaluate the potential prognostic factors for RFS. In univariate analyses, histologic subtype (*p* < 0.001), pathological response (*p* < 0.001), RAR (*p* = 0.025), CEA/albumin ratio (*p* = 0.017), and CA19-9/Albumin ratio (*p* = 0.024) were significantly associated with RFS, whereas age (*p* = 0.056) and Lauren classification (overall *p* = 0.092) demonstrated borderline associations.

Based on these results, variables with univariate *p*-values <0.10 and clinically relevant covariates were entered into the multivariate model. In this analysis, age (HR: 2.417, 95% CI: 1.222–4.780, *p* = 0.011) and histologic subtype (HR: 2.634, 95% CI: 1.356–5.119, *p* = 0.004) were identified as independent predictors of RFS. The Lauren classification (overall *p* = 0.045) also demonstrated a marginal effect. The CEA/albumin ratio, although no longer statistically significant in the multivariate model (*p* = 0.087), showed a trend toward an association with RFS. The RAR and CA19-9/Albumin ratio did not retain significance.

Given its strong association with RFS, the pathological response was analyzed separately and included in a supplementary model. In this analysis, pathological response remained the strongest independent prognostic factor (HR: 0.200, 95% CI: 0.106–0.376, *p* < 0.001), whereas the effects of the other markers were attenuated.

The results of the univariate and multivariate Cox regression analyses are summarized in Table 4, and the supplementary model including pathological response is presented in Supplementary Table S1.

### 3.5. Kaplan–Meier Survival Analyses

**Figure 1:**
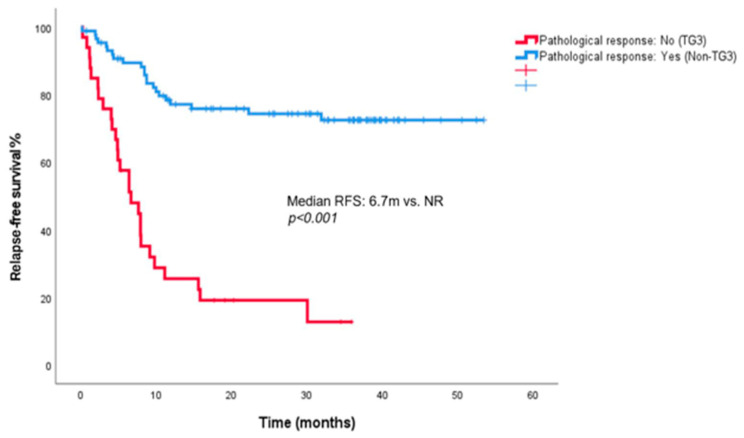
Kaplan–Meier curves for relapse-free survival according to pathological response.

Kaplan–Meier curves demonstrated the prognostic impact of pathological response and CEA/albumin ratio on RFS. Patients without a pathological response had a median RFS of 6.7 months (95% CI, 3.4–10.0), whereas patients with a pathological response did not reach the median RFS (*p* < 0.001) (Figure 1).

**Figure 2:**
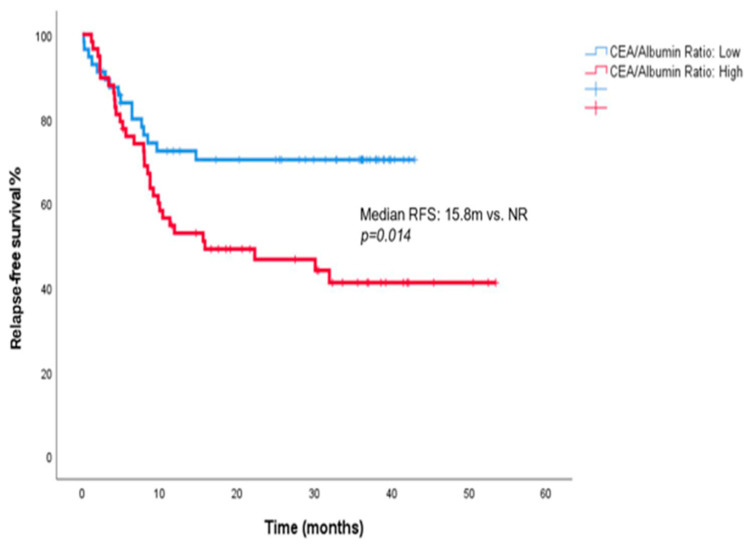
Kaplan–Meier curves for relapse-free survival according to the CEA/albumin ratio.

Similarly, patients with a high CEA/albumin ratio had a median RFS of 15.8 months (95% CI, 0.0–36.6), whereas the median RFS was not reached in those with a low ratio (*p* = 0.014) (Figure 2).

## 4. Discussion

In this study, we evaluated the association between inflammatory and nutritional indices (NLR, PLR, PNI, SII, HALP, NAR, HRR, and RAR) and composite ratios (CEA/albumin and CA19-9/albumin) with both pathological response and relapse-free survival (RFS) in patients with locally advanced gastric cancer who received perioperative FLOT chemotherapy.

Patients who achieved a pathological response showed significantly higher rates of radiologic response and R0 resection, along with lower recurrence rates. This response emerged as the strongest independent prognostic factor for RFS, whereas none of the inflammatory or nutritional indices were significantly associated with the pathological response.

In the RFS analysis, the CEA/albumin ratio trended toward significance but did not reach statistical significance.

Our finding that pathological response emerged as the strongest predictor of RFS aligns with previous studies, highlighting its prognostic significance for treatment efficacy and survival [23,24,27]. However, the findings of studies on inflammatory and nutritional indices have been inconsistent.

Several studies on patients with gastric cancer receiving neoadjuvant chemotherapy have reported significant associations between NLR or PLR and pathological response, whereas others, including ours, did not confirm this association [18,28]. Likewise, some studies have demonstrated a relationship between SII or PNI and pathological response [29,30], while Sugiyama et al. [20] reported that among patients receiving perioperative FLOT, PNI was correlated with RFS but not with pathological response. Such discrepancies may reflect the dynamic nature of systemic inflammation and nutritional status, partly due to the limitation that a single pretreatment measurement may not adequately represent the entire biological response.

Evidence on newer parameters, such as HALP and HRR, is still limited. Köşeci et al. [16] reported that the HALP score was associated with pathological response, whereas Yılmaz et al. [15] found HRR to have prognostic significance. In contrast, our study did not demonstrate a significant association between these indices and pathological response.

There is little data in the literature concerning the impact of CEA/albumin and CA19-9/albumin ratios on pathological response among patients with gastric cancer undergoing neoadjuvant chemotherapy. Some studies have reported an association between these ratios and pathological response [22,31]; however, our cohort did not observe such a relationship.

Overall, the literature on the association between inflammatory and nutritional parameters and pathological response remains inconsistent, particularly in patients receiving FLOT therapy. Our results indicate that these indices may have limited predictive value for the pathological response. Therefore, prospective studies incorporating both pre- and post-treatment assessments are warranted to better define the predictive role of these factors.

In our study, the median follow-up was 26.1 months. The median RFS was not reached, whereas the median overall survival (OS) was 49.1 months. In the multivariate analysis, pathological response remained the most powerful determinant of RFS, while histologic subtype and age also emerged as independent prognostic factors. In univariate analyses, the RAR, CEA/albumin, and CA19-9/albumin ratios were significantly associated with RFS; however, in multivariate analysis, only the CEA/albumin ratio showed a non-significant trend toward association.

Evidence linking inflammatory and nutritional scores with RFS remains limited, particularly in patients who receive neoadjuvant therapy. In patients undergoing surgery without neoadjuvant chemotherapy, NLR has been reported to correlate with RFS [32]; however, this association has not been confirmed in cohorts treated with neoadjuvant chemotherapy [11,18]. These discrepancies may reflect variations in the immune cell responses induced by chemotherapy. Similarly, although PNI and SII have been shown to correlate with RFS in some studies [13,14,15], this association was not observed in another cohort treated with perioperative FLOT [19].

Evidence regarding the HALP score and RFS in the neoadjuvant setting is also limited. Some studies have reported that HALP is prognostic for overall survival rather than RFS [16,33], and a large meta-analysis including solid tumors showed that a low HALP score was significantly associated with poor prognosis [34]. However, in our analysis, the HALP score was not significantly associated with RFS.

Previous studies have shown that serum CEA and CA19-9 levels are associated with both RFS and OS in gastric cancer [35]. However, studies specifically evaluating the prognostic value of the CEA/albumin and CA19-9/albumin ratios remain limited. Our study adds to the literature by assessing these two composite parameters. We found that patients with a low CEA/albumin ratio had significantly longer RFS than those with a high ratio (median RFS: not reached vs. 15.8 months, *p* = 0.014). This suggests that the CEA/albumin ratio may serve as a biomarker for predicting the risk of recurrence in locally advanced gastric cancer treated with perioperative FLOT. However, these findings should be validated in larger, ideally prospective cohorts.

Chronic inflammation plays a central role in the pathogenesis of gastric cancer by remodeling the tumor microenvironment and initiating metastasis. The infiltration of inflammatory cells promotes tumor progression, angiogenesis, and immune evasion through cytokine and chemokine release. The activation of neutrophils and platelets may further enable tumor cells to escape immune surveillance and foster the emergence of chemoresistant clones. Malnutrition impairs immune cell function, reduces treatment tolerance, and negatively affects survival [6,7,8]. Therefore, a simultaneous assessment of inflammatory and nutritional status provides a more integrated understanding of tumor biology and treatment response.

The lack of a significant association between inflammatory and nutritional indices and pathological response in our study may reflect the dynamic and systemic nature of these processes. Fluctuations in cytokine levels, bone marrow suppression, and immune cell redistribution during neoadjuvant chemotherapy may compromise the biological stability of these biomarkers. Furthermore, poor nutritional status, particularly hypoalbuminemia, may reduce T-cell proliferation and cytotoxic activity, thereby weakening antitumor immunity. Thus, the combined evaluation of inflammatory and nutritional markers may better capture both the treatment response and long-term recurrence risk. This combined approach has also been emphasized in previous studies as a means of improving prognostic accuracy [9,10].

Recent evidence highlights that the tumor microenvironment (TME) plays a critical role in shaping the biological behavior and heterogeneity of gastric cancer. In particular, complex interactions between cancer-associated fibroblasts (CAFs) and tumor-infiltrating myeloid cells have been shown to modulate immune responses, promote tumor progression, and contribute to an immunosuppressive milieu. These bidirectional signaling pathways, mediated through cytokines, growth factors, and metabolic crosstalk, may influence tumor proliferation, invasion, and therapeutic resistance. In parallel, accumulating evidence suggests that molecular regulators such as microRNAs are closely involved in gastric cancer pathogenesis by controlling key processes, including cell cycle progression, proliferation, and invasion. For instance, dysregulation of specific microRNAs has been associated with altered tumor growth dynamics and prognosis in gastric cancer [36,37,38]. Taken together, these findings support the notion that systemic inflammatory and nutritional indices may reflect, at least in part, the underlying tumor–host interactions and immune landscape. Therefore, the prognostic value of these indices observed in our study may be explained by their indirect association with the biological activity of the tumor microenvironment.

Although our study did not demonstrate a direct association between these parameters and pathological response, the influence of the inflammatory–nutritional balance on tumor biology warrants further attention. This observation suggests that serial assessments encompassing both pre- and post-treatment periods may clarify the prognostic potential of these dynamic indices in future research.

The clinical relevance of inflammatory and nutritional scores lies in their accessibility, low cost, and noninvasive nature. However, inconsistent results across studies regarding their association with pathological response and survival pose major challenges for clinical implementation. These discrepancies likely arise from the heterogeneity of chemotherapy regimens, timing of measurements, and cut-off definitions. In our ROC analyses, no statistically significant cut-off values were identified, and median-based thresholds were used instead. These findings suggest that, without standardized definitions, these indices may not be suitable for independent use in clinical decision-making.

A major strength of our study is the inclusion of a homogeneous cohort of patients who received perioperative FLOT chemotherapy, minimizing biological variability due to differing regimens. In addition, its multicenter design enabled the inclusion of a large and representative patient cohort. Unlike previous studies, our analysis simultaneously evaluated a broad spectrum of inflammatory and nutritional indices within the same cohort in relation to both pathological response and RFS. This comprehensive approach positions our work as a valuable reference for future multivariate prognostic modeling efforts.

Nevertheless, our study had certain limitations. The retrospective design of this study carries the risk of selection and information bias. The relatively small sample size may have reduced the statistical power for some variables, particularly in the multivariate analyses. Because of the relatively short follow-up period, the median RFS was not reached, and OS data are still immature. Moreover, post-treatment changes in inflammatory and nutritional parameters could not be evaluated, which limited the interpretation of temporal changes. However, given the inconsistent results reported in the literature regarding inflammatory and nutritional indices in gastric cancer, our findings may contribute to the ongoing discussion on prognostic factors in patients treated with neoadjuvant FLOT therapy followed by curative resection.

## 5. Conclusions

Our findings indicate that pathological response is the strongest prognostic factor in patients with locally advanced gastric cancer treated with perioperative FLOT. Although the evaluated inflammatory and nutritional indices provide valuable insights into tumor biology, they are not sufficiently strong to serve as independent prognostic markers.

The significant association between the CEA/albumin ratio and RFS suggests that this ratio may serve as a prognostic biomarker, particularly in patients receiving FLOT chemotherapy. Validation in larger, prospectively designed studies could enhance clinical decision-making.

Overall, for these indices to be implemented in clinical practice, standardization, prospective validation, determination of optimal cut-off values, and assessment of their dynamic changes over time are required.

## Figures and Tables

**Table 1:** Clinicopathological characteristics of the overall cohort and according to pathological response.

Variables	Overall (n = 120)	Non-Responders (n = 33)	Responders (n = 87)	*p*
**Age (years)** Median (range)	59 (26–86)	60 (29–86)	59 (26–78)	0.590
**Gender, n (%)** Male Female	85 (71.4%) 34 (28.6%)	26 (78.8%) 7 (21.2%)	59 (68.6%) 27 (31.4%)	0.271
**ECOG, n (%)** 0 1 2	102 (85.0%) 17 (14.2%) 1 (0.8%)	28 (84.8%) 4 (12.1%) 1 (3.0%)	74 (85.1%) 13 (14.9%) 0 (0.0%)	0.250
**Tumor location, n (%)** Antrum Cardia Corpus GEJ	37 (30.8%) 42 (35.0%) 27 (22.5%) 14 (11.7%)	7 (21.2%) 13 (39.4%) 10 (30.3%) 3 (9.1%)	30 (34.5%) 29 (33.3%) 17 (19.5%) 11 (12.6%)	0.375
**Histological subtype, n (%)** Adenocarcinoma Signet-ring cell	90 (75.0%) 30 (25.0%)	21 (63.6%) 12 (36.4%)	69 (79.3%) 18 (20.7%)	0.077
**Lauren classification, n (%)** Intestinal Diffuse/mixed Unknown	77 (64.2%) 24 (20.0%) 19 (15.8%)	16 (48.5%) 10 (30.3%) 7 (21.2%)	61 (70.1%) 14 (16.1%) 12 (13.8%)	0.082
**cT stage, n (%)** T1–2 T3–4	11 (9.2%) 109 (90.8%)	1 (3.0%) 32 (97.0%)	10 (11.5%) 77 (88.5%)	0.151
**cN stage, n (%)** N0 N+	16 (13.3%) 104 (86.7%)	4 (12.1%) 29 (87.9%)	12 (13.8%) 75 (86.2%)	0.810
**Clinical stage, n (%)** II III	19 (15.8%) 101 (84.2%)	4 (12.1%) 29 (87.9%)	15 (17.2%) 72 (82.8%)	0.493
**Primary G-CSF prophylaxis, n (%)** No Yes	27 (22.5%) 93 (77.5%)	11 (33.3%) 22 (66.7%)	16 (18.4%) 71 (81.6%)	0.080
**Radiological response, n (%)** No Yes	34 (28.3%) 86 (71.7%)	27 (81.8%) 6 (18.2%)	7 (8.0%) 80 (92.0%)	<0.001
**R0 resection, n (%)** No Yes	5 (4.2%) 115 (95.8%)	5 (15.2%) 28 (84.8%)	0 (0.0%) 87 (100.0%)	<0.001
**Relapse, n (%)** No Yes	71 (59.2%) 49 (40.8%)	6 (18.2%) 27 (81.8%)	65 (74.7%) 22 (25.3%)	<0.001

ECOG, Eastern Cooperative Oncology Group; GEJ, gastroesophageal junction; G-CSF, granulocyte-colony stimulating factor.

**Table 2:** Inflammatory and nutritional markers of the overall cohort and according to pathological response.

Variables	Overall Cohort (n = 120) Median (Min–Max)	Non-Responders (n = 33) Median (Min–Max)	Responders (n = 87) Median (Min–Max)	*p*
**NLR**	2.61 (0.70–6.91)	2.67 (0.70–6.91)	2.58 (0.81–6.61)	0.886
**PLR**	138 (48–485)	156 (48–485)	135 (74–399)	0.655
**PNI**	48.5 (31.8–88.5)	48.0 (34.8–70.6)	48.8 (31.8–88.5)	0.527
**SII**	746 (122–3683)	820 (122–3683)	665 (239–2370)	0.318
**HALP**	30.8 (3.26–89.3)	25.6 (4.3–86.7)	30.9 (3.26–89.3)	0.722
**NAR**	1.31 (0.29–3.05)	1.30 (0.58–2.78)	1.31 (0.29–3.05)	0.459
**HRR**	0.75 (0.18–1.23)	0.77 (0.20–1.07)	0.75 (0.18–1.23)	0.616
**RAR**	4.10 (2.00–11.7)	4.53 (2.30–9.55)	4.01 (2.00–11.7)	0.108
**CEA/Albumin Ratio**	0.68 (0.09–57.1)	1.04 (0.20–19.5)	0.63 (0.09–57.1)	0.584
**CA19-9/Albumin Ratio**	2.92 (0.05–12,960)	4.52 (0.11–484.7)	2.19 (0.05–12,960)	0.068

NLR, neutrophil-to-lymphocyte ratio; PLR, platelet-to-lymphocyte ratio; PNI, prognostic nutritional index; SII, systemic immune-inflammation index; HALP, hemoglobin–albumin–lymphocyte–platelet score; NAR, neutrophil-to-albumin ratio; HRR, hemoglobin-to-red cell distribution width ratio; RAR, red cell distribution width-to-albumin ratio; CEA, carcinoembryonic antigen; CA19-9, carbohydrate antigen 19-9.

**Table 3:** Univariate and multivariate logistic regression analyses of factors associated with pathological response.

Variable	Univariate OR (95% CI)	*p*	Multivariate OR (95% CI)	*p*
**Age (≥65 vs. <65 years)**	0.667 (0.285–1.561)	0.350		
**ECOG (≥1 vs. 0)**	0.977 (0.321–3.013)	0.977		
**Sex (Male vs. Female)**	0.588 (0.227–1.522)	0.274		
**Treatment center** Cerrahpaşa Medical Faculty Istanbul Training and Research Hospıtal Tekirdağ Namık Kemal Medical Faculty Haseki Training and Research Hospıtal	Reference 7.2 (0.62–83.34) 3.2 (0.75–13.4) 0.97 (0.21–4.3)	0.11 0.11 0.97		
**Tumor location** Cardia vs. Antrum Corpus vs. Antrum GEJ vs. Antrum	0.608 (0.145–2.554) 0.464 (0.104–2.071) 1.169 (0.256–5.337)	0.386 0.497 0.314 0.840		
**Histologic subtype** Signet-ring cell vs. adenocarcinoma	0.457 (0.190–1.099)	0.080	0.383 (0.131–1.124)	0.081
**Lauren classification** Diffuse/mixed vs. Intestinal Unknown vs. Intestinal	2.224 (0.753–6.566) 0.817 (0.237–2.811)	0.089 0.148 0.748	1.579 (0.493–5.059) 0.357 (0.100–1.270)	0.129 0.445 0.112
**cT stage (T3–4 vs. T1–2)**	0.241 (0.030–1.958)	0.183	0.656 (0.071–6.049)	0.729
**cN stage (N+ vs. N0)**	0.862 (0.257–2.891)	0.810		
**Clinical stage (III vs. II)**	0.662 (0.203–2.164)	0.495		
**Primary G-CSF prophylaxis**	2.219 (0.898–5.482)	0.084	2.523 (0.916–6.951)	0.073
**NLR (High vs. Low)**	0.920 (0.413–2.050)	0.838		
**PLR (High vs. Low)**	0.657 (0.293–1.474)	0.308		
**PNI (High vs. Low)**	1.286 (0.575–2.873)	0.540		
**SII (High vs. Low)**	0.657 (0.293–1.474)	0.308		
**HALP (High vs. Low)**	1.087 (0.488–2.424)	0.838		
**NAR (High vs. Low)**	0.963 (0.432–2.147)	0.927		
**HRR (High vs. Low)**	0.858 (0.384–1.915)	0.708		
**RAR (High vs. Low)**	0.474 (0.207–1.084)	0.077	0.883 (0.672–1.163)	0.362
**CEA/Albumin (High vs. Low)**	0.729 (0.315–1.688)	0.461		
**CA19-9/Albumin (High vs. Low)**	0.489 (0.207–1.154)	0.103	0.519 (0.202–1.333)	0.173

ECOG, Eastern Cooperative Oncology Group; OR, odds ratio; CI, confidence interval; NLR, neutrophil-to-lymphocyte ratio; PLR, platelet-to-lymphocyte ratio; PNI, prognostic nutritional index; SII, systemic immune-inflammation index; HALP, hemoglobin–albumin–lymphocyte–platelet score; NAR, neutrophil-to-albumin ratio; HRR, hemoglobin-to-red cell distribution width ratio; RAR, red cell distribution width-to-albumin ratio; CEA, carcinoembryonic antigen; CA19-9, carbohydrate antigen 19-9.

**Table 4:** Univariate and multivariate Cox regression analyses of clinicopathological variables and inflammatory/nutritional markers associated with RFS.

Variable	Univariate HR (95% CI)	*p*	Multivariate HR (95% CI)	*p*
**Age (≥65 vs. <65 years)**	1.751 (0.985–3.113)	0.056	2.417 (1.222–4.780)	0.011
**Sex (Male vs. Female)**	1.039 (0.559–1.932)	0.903		
**ECOG (≥1 vs. 0)**	1.379 (0.668–2.847)	0.385		
**Tumor location** Cardia vs. Antrum Corpus vs. Antrum GEJ vs. Antrum	1.143 (0.459–2.846) 0.854 (0.316–2.312) 0.673 (0.253–1.794)	0.530 0.775 0.757 0.429		
**Histologic subtype** Signet-ring cell vs. adenocarcinoma	3.296 (1.866–5.822)	<0.001	2.634 (1.356–5.119)	0.004
**Lauren classification** Diffuse/mixed vs. Intestinal Unknown vs. Intestinal	0.559 (0.262–1.194) 1.083 (0.462–2.538)	0.092 0.133 0.855		0.045
**cT stage (T3–4 vs. T1–2)**	2.076 (0.645–6.681)	0.221		
**cN stage (N+ vs. N0)**	1.605 (0.636–4.048)	0.317		
**Clinical stage (III vs. II)**	1.425 (0.639–3.175)	0.386		
**Primary G-CSF prophylaxis (Yes vs. No)**	0.629 (0.338–1.172)	0.145		
**Pathological response (Yes vs. No)**	0.169 (0.095–0.301)	<0.001		
**NLR (High vs. Low)**	1.089 (0.622–1.908)	0.765		
**PLR (High vs. Low)**	1.229 (0.701–2.155)	0.473		
**PNI (High vs. Low)**	0.789 (0.450–1.383)	0.408		
**SII (High vs. Low)**	1.204 (0.687–2.110)	0.516		
**HALP (High vs. Low)**	1.181 (0.672–2.074)	0.563		
**NAR (High vs. Low)**	1.174 (0.669–2.059)	0.576		
**HRR (High vs. Low)**	1.416 (0.800–2.504)	0.233		
**RAR (High vs. Low)**	1.954 (1.088–3.509)	0.025	1.476 (0.777–2.804)	0.234
**CEA/Albumin Ratio (High vs. Low)**	2.082 (1.141–3.800)	0.017	1.805 (0.918–3.551)	0.087
**CA19-9/Albumin Ratio (High vs. Low)**	1.989 (1.095–3.614)	0.024	1.491 (0.749–2.968)	0.255

ECOG, Eastern Cooperative Oncology Group; HR, hazard ratio; CI, confidence interval; NLR, neutrophil-to-lymphocyte ratio; PLR, platelet-to-lymphocyte ratio; PNI, prognostic nutritional index; SII, systemic immune-inflammation index; HALP, hemoglobin–albumin–lymphocyte–platelet score; NAR, neutrophil-to-albumin ratio; HRR, hemoglobin-to-red cell distribution width ratio; RAR, red cell distribution width-to-albumin ratio; CEA, carcinoembryonic antigen; CA19-9, carbohydrate antigen 19-9.

## Data Availability

The datasets generated and/or analyzed during the current study are available from the corresponding author.

## Supplementary Materials

The following supporting information can be downloaded at https://www.mdpi.com/article/10.3390/jcm15072574/s1, Figure S1: Kaplan–Meier survival curve demonstrating overall survival in the study cohort; Table S1: Univariate and multivariate Cox regression analysis for overall survival.

## Abbreviations

The following abbreviations are used in this manuscript:

AJCC	American Joint Committee on Cancer
AUC	Area under the curve
CA19-9	Carbohydrate antigen 19-9
CEA	Carcinoembryonic antigen
CI	Confidence interval
CRP	C-reactive protein
RFS	Relapse-free survival
ECOG	Eastern Cooperative Oncology Group
FLOT	Fluorouracil, leucovorin, oxaliplatin, and docetaxel
G-CSF	Granulocyte-colony stimulating factor
HALP	Hemoglobin–albumin–lymphocyte–platelet score
HR	Hazard ratio
HRR	Hemoglobin-to-red cell distribution width ratio
NAR	Neutrophil-to-albumin ratio
NLR	Neutrophil-to-lymphocyte ratio
OR	Odds ratio
OS	Overall survival
PNI	Prognostic nutritional index
PLR	Platelet-to-lymphocyte ratio
R0	Microscopically margin-negative resection
R1	Microscopically margin-positive resection
RAR	Red cell distribution width-to-albumin ratio
RDW	Red cell distribution width
ROC	Receiver operating characteristic
SII	Systemic immune-inflammation index
SPSS	Statistical Package for the Social Sciences
TRG	Tumor regression grade
