# Supplementary material for: Inflammatory and Nutritional Indices as Prognostic Markers in Locally Advanced Gastric Cancer Treated with Neoadjuvant FLOT: A Retrospective Multicenter Study
Source: J Clin Med. 2026 Mar 27;15(7):2574. doi: 10.3390/jcm15072574 (PMC13073396; doi:10.3390/jcm15072574)
Supplement: Supplementary file 1 [file jcm-15-02574-s001.zip › jcm-4178830-supplementary.pdf]

Figure S1. Kaplan–Meier survival curve demonstrating overall survival in the study cohort. During follow-up, 49 patients died. The median overall survival was 49.0 months (95% CI: 40.15–57.85). Tick marks on the curve represent censored observations.

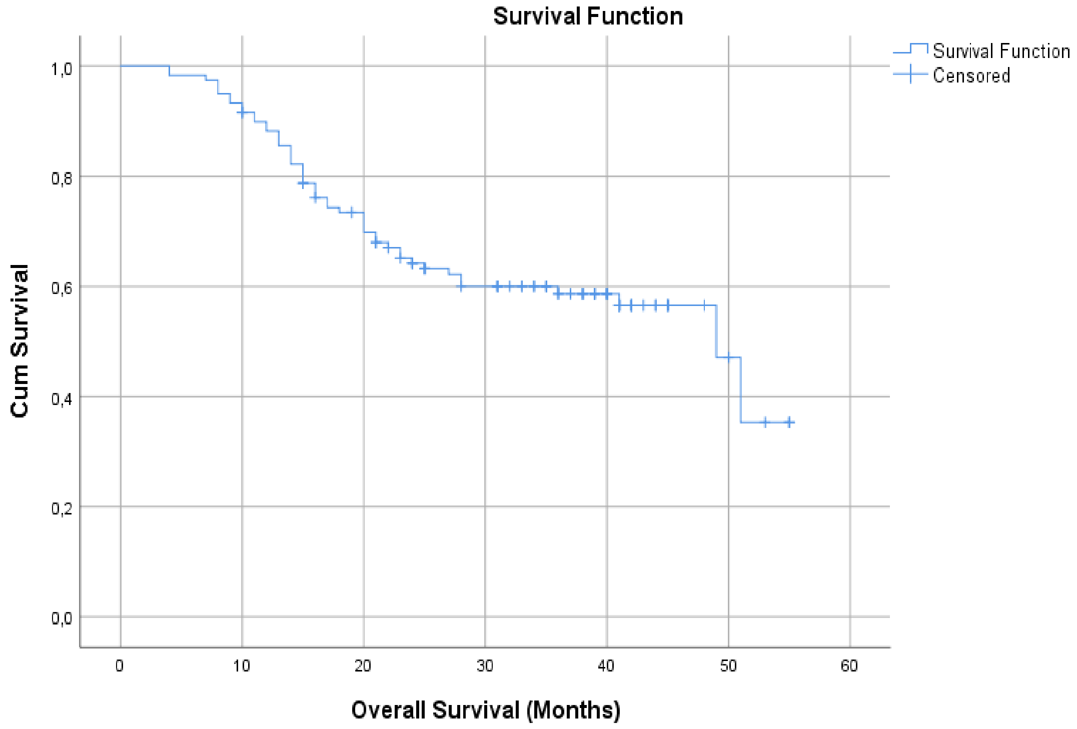

Table S1. Univariate and multivariate Cox regression analysis for overall survival

Variable	Univariate HR (95% CI)	p	Multivariate HR (95% CI)	P
Age (≥65 vs. <65 years)	1.3 (0.73-2.29)	0.36		
Sex (Male vs. Female)	1.09 (0.58-2.04)	0.77		
ECOG (≥1 vs. 0)	1.90 (1.00-3.6)	0.04	2.08 (1.05-4.13)	0.03
Histologic subtype<br>Signet-ring cell vs adenocarcinoma	1.48 (1.10-1.99)	<0.009	3.08 (1.64-5.79)	<0.001
cT stage (T3–4 vs. T1–2)	3.26 (0.79-13.45)	0.10		
cN stage (N+ vs. N0)	1.42 (0.56-3.59)	0.45		
Pathological response (Yes vs. No)	0.2 (0.11-0.36)	<0.001	0.21 (0.11-0.4)	<0.001
